# Prognostic nomogram in patients with epithelioid sarcoma: A SEER‐based study

**DOI:** 10.1002/cam4.5230

**Published:** 2022-09-08

**Authors:** Di Zhang, Jintao Hu, Zhuojie Liu, Haoyu Wu, HanWen Cheng, Chunhai Li

**Affiliations:** ^1^ Department of Orthopedics, Sun Yat‐sen Memorial Hospital Sun Yat‐sen University Guangzhou China; ^2^ Department of Urology, Sun Yat‐sen Memorial Hospital Sun Yat‐sen University Guangzhou China

**Keywords:** epithelioid sarcoma, nomogram, prognostic model, SEER

## Abstract

**Objective:**

The prognostic factors for patients with epithelial sarcoma remain unclear. The study aims to develop a practical clinical nomogram that predicts prognosis in patients with ES using the Surveillance, Epidemiology, and End Results (SEER) database.

**Methods:**

We extracted clinical data from 2004 to 2015 from the SEER database about patients with ES. All patients were randomly divided into training cohort and validation cohort. Kaplan–Meier analyses were used to compare outcomes between different subgroups. In order to estimate the chance of survival for patients with ES, we developed a nomogram. Nomogram performance was evaluated by discrimination and calibration. Additionally, an analysis of decision curves was conducted to evaluate the clinical usefulness of this newly developed model.

**Results:**

In the primary cohort,320 met the inclusion criteria to be entered into this study. The median OS was 66.000 months (range 34.704 to 94.296 months), and the 1‐, 3‐, and 5‐year OS rates were 70.7%, 56.1%, and 50.4%, respectively. For the validation cohort, we studied 136 consecutive patients. Age, primary site, grade, AJCC (American Joint Committee on Cancer) T, AJCC M, and surgery were included in the nomogram. The C‐index values for the training set and validation set were 0.817 and 0.832, respectively. The calibration plots showed good agreement between the prediction and the observation. Based on the clinical decision curve, the model has a good clinical net benefit for ES patients.

**Conclusions:**

It is the first study that developed an effective survival prediction model for patients with ES. Using this nomogram can assist in clinical decision‐making as it has satisfactory accuracy. Even so, additional external validation is needed.

## Graphical abstract

It is the first study that develope an effective survival prediction model for patients with ES. Using this nomogram can assist in clinical decision‐making as it has satisfactory accuracy. Even so, additional external validation is needed.

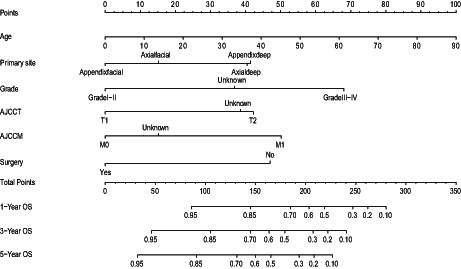

## 1 INTRODUCTION

Epithelioid sarcoma (ES) is a rare subtype of soft tissue sarcoma with highly malignant first described by Enzinger in 1970.^1^, ^2^ It is thought that the occurrence of ES is correlated with the loss of expression of the SMARCB1/INI1 protein.^3^, ^4^ It was found to account for less than 1% of all soft tissue sarcomas.^5^ Despite its slow growth, the tumor is prone to recurrence postoperatively and lymphatic system metastasis. Epithelioid sarcoma has an unfavorable prognosis in general. According to previous reports, the 5‐year overall survival rate ranged from 32 to 92%.^5^, ^6^, ^7^, ^8^, ^9^, ^10^, ^11^, ^12^, ^13^, ^14^ ES typically occurs in young males, predominantly in the distal part of the upper extremity. Surgical resection is the main treatment for ES in many cases. Study have been conducted to investigate the efficacy of surgery, out of 23 patients diagnosed with ES, 16 had a significantly better prognosis after radical surgery than the 7 who underwent conservative treatment.^5^ Recent studies have shown that Tazemetostat is effective in treating epithelioid sarcoma, whereas chemotherapy and radiotherapy have limited effect on sarcoma. Although there are studies exploring the prognostic factors of the disease, there is no prognostic model that can be applied to clinical work to provide a quantitative assessment of patient prognosis.

## 2 METHODS

### 2.1 Data source

The SEER database contains millions of patient records, which encompass a period ranging from 1973 to 2018. These records represent over 30% of the total U.S. population. All cases were obtained from 18 local cancer registries. We attempt to find the independent risk factors affecting prognosis and develop a nomogram to predict the survival of patients with ES based on clinicopathological data. We used the SEER database to identify all cases of ES diagnosed between 2004 and 2015 based on ICD‐O‐3.^15^ Inclusion criteria were as follows: (1) Patients diagnosed with epithelioid sarcoma in the SEER database and (2) the years of diagnosis ranged from 2004 to 2015. Exclusion criteria were as follows: (1) Patients with a history of other cancer; (2) no evidence of primary tumor; and (3) patients with unknown information of primary site, chemotherapy, survival months, or other important demographic, clinical, pathologic, and treatment variables. Patients with ES who met the criteria were randomly assigned to training cohorts and validation cohorts. We collected the following information: age, race, gender, tumor location, tumor size, T stage, N stage, M stage, survival time, and survival status. The racial categories included black, white, and others. We classified the primary site as superficial appendicular locations, deep appendicular locations, superficial axial locations, and deep axial locations. In terms of laterality, three categories can be distinguished: right‐origin of primary, left‐origin of primary, and others. Grade is divided into the following categories: G1‐G2, G3‐G4, and unknown. Furthermore, In the T stage, there were T1, T2, and TX. According to the 6th edition of the AJCC staging system, the T0 stage means no evidence of primary tumor, and these patients were excluded from this study. N0, N1, and Nx constitute the N stage. No metastasis is indicated by M0, positive metastasis by M1, and unknown by MX. One‐, three‐, and five‐year overall survival (OS) rates were the study endpoints.

### 2.2 Statistical analysis

A patient's survival time is defined as the period between the time of diagnosis until the last follow‐up or death. Statistical significance is defined as *p* < 0.05 in two‐sided analyses. Survival curves are calculated using Kaplan–Meier method. Based on the log‐rank test, we evaluated the survival differences between the subgroups. To identify prognostic variables, the Cox regression model with hazard ratios (HRs) and 95% confidence intervals (CIs) was used in the training cohort. A multivariate analysis using forward stepwise regression was conducted using variables selected in univariate regression with a *p*‐value < 0.05. We chose T, N, and M variables instead of stage variables to avoid multicollinearity in the multivariate analysis. Identification and calibration measurements were used to verify the nomogram model. We calculated the C‐index, which quantifies the difference between observations and predictions, and shows the predictive power of the model. ROC curves (Receiver operating characteristic curve) and calibration curves are plotted to verify the discrimination and calibration of the model. The calibration plot of the model displays the calibration between the predicted and actual rates of survival. Additionally, decision curve analysis (DCA) was performed to evaluate the clinical effectiveness and benefit of the prediction model. We used IBM SPSS Statistics 23 and R software (version 4.0.3) for our statistical analyses.

## 3 RESULT

### 3.1 Patient baseline characteristics

From 2004 to 2015, SEER recorded 622 patients with ES. Our analysis involved 456 patients (320 in the development group and 136 in the validation group; Table 1) who met the research criteria. Table 1 shows the baseline characteristics of the study population. There was complete information on the survival times and causes of death of all patients. In the training group, the 1‐year, 3‐year, and 5‐year overall survival rates were 70.7%, 56.1%, and 50.4%. The mean age was 44.73. A large majority of patients are white, around 77.7%. The proportion of females is smaller than that of males. More than half of patients (60.9%) with sarcoma have metastases distant from the primary tumor. A total of 237 cases (70.4%) were surgically treated.

**TABLE 1:** Baseline characteristics of the study population

Variable	Primary cohort (*n* = 320)		Validation cohort (*n* = 136)			*p*‐value
	No. of patients	%	No. of patients		%	
Age, years^a^						0.310
Mean		44.730			42.570	
*SD*		20.300			21.930	
Sex^b^						0.020
Male	184	57.500	62		45.590	
Female	136	42.500	74		54.410	
Race^b^						0.315
White	251	77.680	106		77.940	
Black	37	12.840	21		15.440	
Other	32	9.480	9		6.620	
Laterality^b^						0.465
Right—origin of primary	118	36.880	42		30.880	
Left—origin of primary	84	26.250	40		29.410	
Other	118	36.880	54		39.710	
Primary site^c^						0.209
Superficial axial	96	30.000	28		20.590	
Deep axial	60	18.750	28		20.590	
Superficial appendicular	158	49.380	77		56.620	
Deep appendicular	6	1.880	3		2.210	
Grade^b^						0.247
Grades I‐II	24	7.500	7		5.150	
Grades III‐IV	132	41.250	67		49.260	
Unknown	164	51.250	62		45.590	
AJCC T^b^						0.932
T1	100	31.250	44		32.350	
T2	159	49.690	68		50.000	
Unknown	61	19.060	24		17.650	
AJCC N^b^						0.881
N0	196	61.250	82		60.290	
N1	37	11.560	18		13.240	
Unknown	87	27.190	36		26.470	
AJCC M^b^						0.810
M0	195	60.940	82		60.290	
M1	55	17.190	21		15.440	
Unknown	70	21.880	33		24.260	
Surgery^b^						0.588
Yes	237	74.060	104		76.470	
No	83	25.940	32		23.530	
Radiation^b^						0.904
Yes	129	40.310	54		39.710	
No	191	59.690	82		60.290	
Chemotherapy^b^						0.900
Yes	96	30.000	40		29.410	
No	224	70.000	96		70.590	
Survival months^a^						0.350
Mean		54.480				
*SD*		52.110				
Vital status^b^						0.923
Alive	149	46.560	64		47.060	
Death	171	53.440	72		52.940	

Abbreviation: AJCC, American Joint Committee on Cancer.

^a^ Independent samples *t*‐test.

^b^ Chi‐square test.

^c^ Fisher's precision probability test.

### 3.2 Univariate and multivariate cox regression analysis

Results of univariate and multivariate Cox regression for OS were stated in Table 2. Kaplan–Meier survival curves were plotted for the five categorical variables of primary site, grade, AJCC T, AJCC M, and surgery. The differences in the ES survival time distributions were examined using the log‐rank method. (Figure 5) The differences in the overall survival time distributions between groups for these variables were statistically significant (*p* < 0.05).The variables in the univariate Cox regression that were statistically significant (*p* < 0.05) were incorporated into the multivariate analysis. In the multivariate analysis of OS, variables including age, primary site, grade, AJCC T, AJCC M, and surgery were all statistically significant. According to multivariate analysis, the outcomes were improved in patients with younger age, superficial Primary site, well‐differentiated stage, lower T and M stage, surgery.

**TABLE 2:** Univariate and multivariate Cox analyses of patients with epithelioid sarcoma

Variable	Univariate analysis			Multivariate analysis		
	HR	95% CI	*p*‐value	HR	95% CI	*p*‐value
Age	1.032	1.024–1.041	<0.001	1.022	1.014–1.031	<0.001
Sex			0.310			
Male	1(ref)					
Female	0.854	0.628–1.160	0.311			
Primary site			<0.001			0.004
Superficial axial	1(ref)			1(ref)		
Deep axial	2.208	1.486–3.279	<0.001	1.637	1.045–2.564	0.031
Superficial appendicular	0.562	0.392–0.805	0.002	0.742	0.513–1.074	0.113
Deep appendicular	1.194	0.432–3.299	0.733	1.670	0.587–4.754	0.336
Race			0.560			
White	1(ref)					
Black	1.176	0.748–1.848	0.483			
Other	1.248	0.771 –2.021	0.367			
Grade			<0.001			<0.001
Unknown	1(ref)			1(ref)		
Grades I‐II	0.445	0.194–1.023	0.057	0.486	0.206–1.148	0.100
Grades III‐IV	2.039	1.495–2.781	<0.001	1.841	1.313–2.581	<0.001
Laterality			<0.001			
Right—origin of primary	1(ref)					
Left—origin of primary	0.506	0.325–0.786	0.002			
Other	1.596	1.146–2.223	0.006			
AJCC T			<0.001			0.003
T1	1(ref)			1(ref)		
T2	5.157	3.253–8.173	<0.001	2.325	1.385–3.905	0.002
Unknown	4.034	2.391–6.807	<0.001	2.162	1.224–3.817	0.009
AJCC N			<0.001			
N0	1(ref)					
N1	1.843	1.171–2.901	0.008			
Unknown	2.137	1.536–2.974	<0.001			
AJCC M			<0.001			<0.001
M0	1(ref)			1(ref)		
M1	5.996	4.149–8.666	<0.001	2.668	1.759–4.046	<0.001
Unknown	2.311	1.592–3.356	<0.001	1.345	0.862–2.101	0.192
Surgery			<0.001			<0.001
No	1(ref)			1(ref)		
Yes	0.224	0.164–0.305	<0.001	0.399	0.276–0.575	<0.001
Radiation			0.598			
No	1(ref)					
Yes	1.081	0.797 –1.465	0.616			
Chemotherapy			<0.001			
No	1(ref)		<0.001			
Yes	2.549	1.877–3.461	<0.001			

Abbreviation: AJCC, American Joint Committee on Cancer.

### 3.3 Construction and validation of prognostic nomogram

**FIGURE 1:**
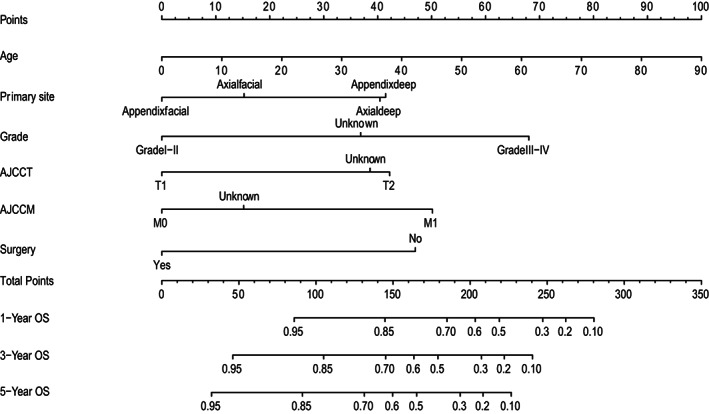
Nomogram predicting 1‐, 3‐, and 5‐year OS for patients with ES.

**FIGURE 2:**
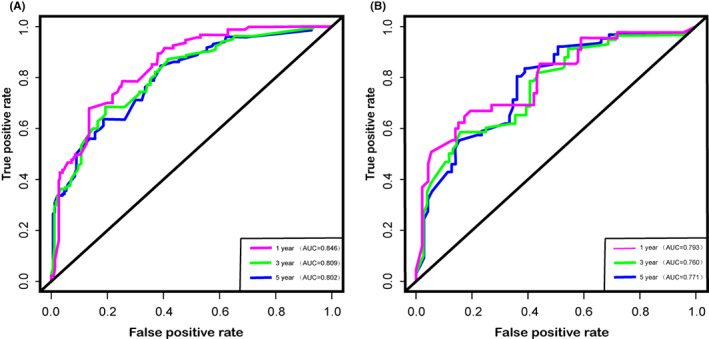
Receiver operating characteristic curve analysis for evaluating the accuracy of the 1‐, 3‐, and 5‐year nomogram. (A) training group and (B) validation group.

**FIGURE 3:**
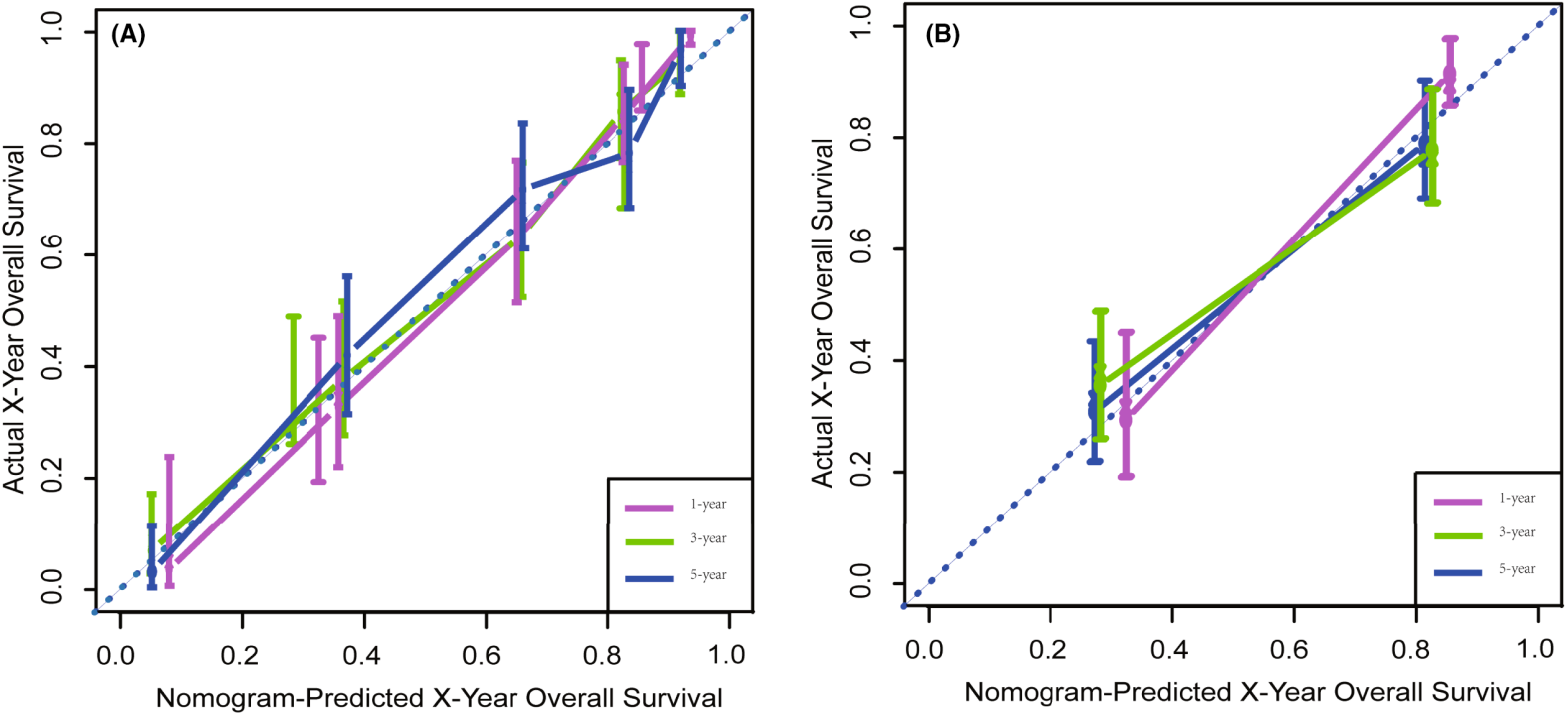
Calibration plots of the nomogram for predicting 1‐, 3‐, and 5‐year OS. Nomogram‐predicted OS is plotted on the x‐axis; actual OS is plotted on the y‐axis. (A) Training group and (B) validation group.

**FIGURE 4:**
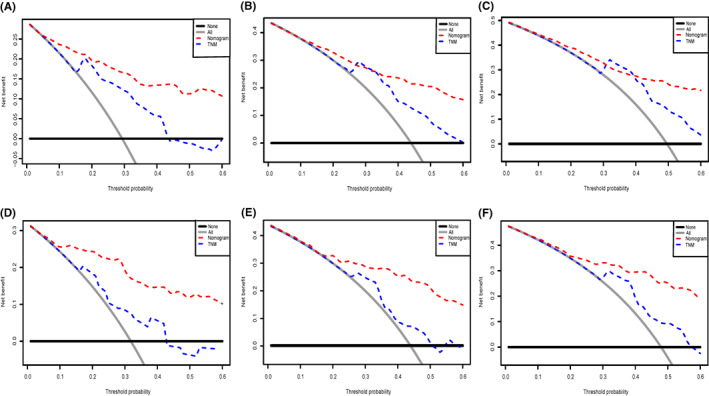
Decision curve analysis for evaluating the net benefit of nomogram and 6th AJCC TNM grading system. (A) 1‐year net benefit in training cohort; (B) 3‐year net benefit in training cohort; (C) 5‐year net benefit in training cohort; (D) 1‐year net benefit in validation cohort; (E) 3‐year net benefit in validation cohort. 5‐year net benefit in validation cohort.

We depict the model incorporating six independent factors that affect overall survival. (Figure 1) The C‐index of this model was 0.82, which represents an ideal correspondence between the predictions and the observed outcomes for survival at 1, 3, and 5 years. ROC plots were generated using the “survivalROC” package for the training and validation group at different times, with the death rate expressed as a continuous variable. AUC was calculated for each plot. (Figure 2) In the training set, the prognostic model accurately predicted 1‐, 3‐, and 5‐year mortality with AUCs of 0.846, 0.809, and 0.802, respectively. In the validation set, AUC values at 1, 3, and 5 years were 0.793, 0.760, and 0.771, respectively. The result shows this model is highly accurate. Figure 3 showed the calibration plots of the nomogram. The DCA results indicate the model provides good net benefits to ES patients. (Figure 4).

**FIGURE 5:**
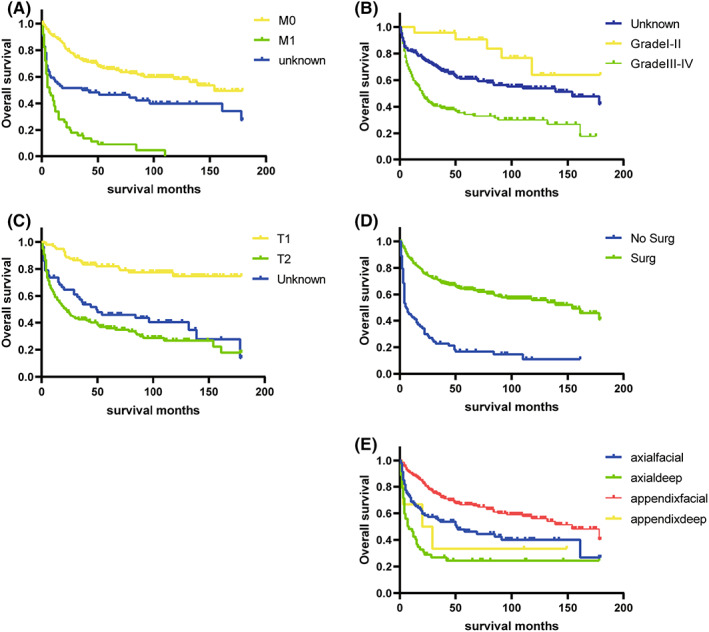
OS for the ES patients using Kaplan–Meier analysis and log‐rank test. (A) OS among ES patients based on M0 stage, M1 stage, and unknown M stage. (B) OS among ES patients based on G1‐G2, G3‐G4, and unknown grades. (C) OS among ES patients based onT1 T2 and unknown T stage. (D) OS among ES patients based on surgery and no surgery. (E) OS among ES patients based on superficial appendicular locations; deep appendicular locations; superficial axial locations; and deep axial locations.

## 4 DISCUSSION

According to our knowledge, no existing epithelioid sarcoma prognosis prediction nomogram has been developed and validated before this study. Nomograms rely on easy‐to‐use digital interfaces, better accuracy, and more clear prognoses to help doctors make better decisions. Currently, nomograms are widely used in clinical work as prognostic devices. The prognostic predictors for the nomogram were age, primary tumor site, grade, surgery, T stage, and M stage. (Figure 1).

In terms of the ability to predict the prognosis of patients, we compared our prediction model with the TNM staging system. In training and validation cohorts, the C‐index was 0.818 and 0.832 for predicting OS, while the C‐index for TNM stage was 0.744 and 0.724. In terms of predicting the 1‐year, 3‐year, and 5‐year OS, the areas under the curves of our model are 0.846, 0.809, and 0.802, respectively, while the areas under the curves of TNM stages are 0.838, 0.777, and 0.784, respectively. From the results of the above, we can easily find that our model based on more clinical information has a better predictive effect than the TNM staging system.

Using the SEER database, we created a prognostic model to determine the prognosis of ES patients based on large‐sample data. A total of 456 patients were enrolled in this study. This study supports previous research that elderly patients have a poorer prognosis.^16^ It may be due to the fact that elderly patients tend to have multiple comorbid conditions, such as coronary artery disease and hypertension, which are associated with a worse prognosis. Additionally, primary tumor site was identified as an independent factor for prognosis (*p* < 0.05). According to a previous study by Livi et al.^17^ patients with tumor sites distal to the body have a better prognosis. This study revealed that tumors deepen into the fascia have a worse prognosis, which is in line with previous studies.^13^ Previous studies have suggested that deep tumor localization is an independent risk factor for local recurrence‐free survival.^7^ It is possible to hypothesize that ES with deep‐seated location may be difficult to diagnose because these patients do not have obvious symptoms.^9^ Conversely, Ross et al^18^ reported that the primary tumor site had no impact on patient survival. There has previously been debate about grading for ES.^19^, ^20^ In our study, the grade was significant in both univariate and multivariate regression analyses and was an independent risk factor. However, there were far fewer patients in the validation and training cohort at the lower grade than at the higher grade. Results, therefore, need to be interpreted with caution. The majority of cases with reported values were high grade. Thus, it may help explain why ES has such a poor prognosis. T stage and M stage are independent risk factors for OS. Studies on the topic indicate that tumors larger than 5 cm tend to have worse outcomes.^2^, ^21^ Tumors that measure more than 5 cm should receive adjuvant radiation.^18^ The study by Spillane et al^13^ reported that there was a significant improvement in distant metastasis‐free survival in smaller ES (<5 cm) but the size was not a significant predictor of overall survival. Some scholars also propose that the multifocality of ES lesions prevents precise measurements of tumor size.^7^ Prognosis is usually less favorable for patients with distant metastases of ES at the time of diagnosis.^5^, ^13^, ^22^ A high number of distant metastases have been observed in patients with ES, which range from 40% to 57%,^5^, ^7^, ^13^, ^18^ and these metastases may be resistant to intensive treatment. Our analyses indicated that patients with localized and regional ES fared better than those with distant metastases. Although ES has a high rate of metastatic spread to regional lymph nodes, ranging from 10% to 65% in the literature,^5^, ^10^, ^18^, ^22^, ^23^, ^24^ N stage did not contribute significantly to OS in our study. Previous studies have mentioned that nodal metastasis resection is not very effective in extending the survival time.^25^ The current studies support the point of previous research that surgical interventions had a positive impact on patient outcomes.^5^, ^14^ In the past, surgical treatment including amputation has always been the mainstay of ES patients. Recently, researchers have found that Tazemetostat has the potential to improve outcomes in patients with advanced ES in a prospective clinical trial of 62 patients.^26^ Tazemetostat has been approved by the FDA for treating epithelioid sarcoma. Targeted treatment for epithelioid sarcoma, this drug is the first of its kind.^27^ With the development of targeted drugs, ES patients will have access to more treatment options.

Our research shows that the model efficiently predicts the survival of patients with ES, thereby improving the accuracy of clinical decision‐making. There are some limitations to our study that should be noted. Firstly, stages were organized based on the 6th AJCC staging system, which could limit its effectiveness. Secondly, our nomogram was constructed based on the SEER database, where partial patient information is necessarily lost. As a result, there may be fewer qualified cases, which may increase the selection bias. Lastly, the study is a retrospective cohort study. In the current situation, given that ES is a very rare soft tissue sarcoma, the most reliable way to study the prognosis of this disease is using public database. Yet, the prognostic nomogram we constructed needs to be validated by further prospective cohort studies.

## 5 CONCLUSION

It is the first study that developed an effective survival prediction model for patients with ES. Using this nomogram can assist clinical decision‐making as it has satisfactory accuracy. Even so, additional external validation is needed.

## AUTHOR CONTRIBUTION

Study design: DZ, JH, and ZL. Methodological development: DZ and JH. Data acquisition and statistical analysis: DZ, JH, ZL, HW, and HC. Study Research guidance and supervision: CL. All authors contributed to the article and approved the submitted version, and wrote the manuscript.

## FUNDING INFORMATION

This work was supported by the Science and Technology Program of Guangzhou(202102010259).

## CONFLICT OF INTEREST

The authors declare that they have no conflicts of interest related to this paper.

## ETHICS STATEMENT

No personal identifying information was used in the study. Hence, we did not require Institutional Review Board approval or patient informed consent.

## AUTHORSHIP CLARIFIED

All authors agreed with the content, all gave explicit consent to submit, and we obtained consent from the responsible authorities at the institution where the work has been carried out, before the work is submitted.

## Data Availability

All data were downloaded from the SEER (https://seer.cancer.gov/). Data from this study are available to any interested researchers upon reasonable request to the corresponding author.
